# B-cell Receptor Signaling Induced Metabolic Alterations in Chronic Lymphocytic Leukemia Can Be Partially Bypassed by *TP53* Abnormalities

**DOI:** 10.1097/HS9.0000000000000722

**Published:** 2022-05-13

**Authors:** Katarina Kluckova, Andrew J. Clear, Annalisa D’Avola, Laura Z. Rassenti, Thomas J. Kipps, John G. Gribben, John C. Riches

**Affiliations:** 1 Centre for Haemato-Oncology, Barts Cancer Institute—A Cancer Research UK Centre of Excellence, Queen Mary University of London, United Kingdom; 2 The Francis Crick Institute, London, United Kingdom; 3 Moores Cancer Center, University of California, San Diego, La Jolla, CA, USA

## Abstract

It has been unclear what role metabolism is playing in the pathophysiology of chronic lymphocytic leukemia (CLL). One reason is that the study of CLL metabolism is challenging due to the resting nature of circulating CLL cells. Also, it is not clear if any of the genomic aberrations observed in this disease have any impact on metabolism. Here, we demonstrate that CLL cells in proliferation centers exhibit upregulation of several molecules involved in glycolysis and mitochondrial metabolism. Comparison of CXCR4/CD5 intraclonal cell subpopulations showed that these changes are paralleled by increases in the metabolic activity of the CXCR4^low^CD5^high^ fraction that have recently egressed from the lymph nodes. Notably, anti-IgM stimulation of CLL cells recapitulates many of these metabolic alterations, including increased glucose uptake, increased lactate production, induction of glycolytic enzymes, and increased respiratory reserve. Treatment of CLL cells with inhibitors of B-cell receptor (BCR) signaling blocked these anti-IgM-induced changes in vitro, which was mirrored by decreases in hexokinase 2 expression in CLL cells from ibrutinib-treated patients *in vivo.* Interestingly, several samples from patients with 17p-deletion manifested increased spontaneous aerobic glycolysis in the unstimulated state suggestive of a BCR-independent metabolic phenotype. We conclude that the proliferative fraction of CLL cells found in lymphoid tissues or the peripheral blood of CLL patients exhibit increased metabolic activity when compared with the bulk CLL-cell population. Although this is due to microenvironmental stimulatory signals such as BCR-engagement in most cases, increases in resting metabolic activity can be observed in cases with 17p-deletion.

## INTRODUCTION

Deregulation of cellular energetics is an established hallmark of cancer.^1^ One of the most important observations regarding tumor metabolism was made by Otto Warburg in 1924: cancer cells take up and preferentially metabolize glucose to lactate even in the presence of oxygen (aerobic glycolysis). The reason for this was initially unclear, as this is an inefficient way of producing energy, but it is now apparent that this “Warburg effect” enables cancer cells to use glucose as a substrate for the production of nucleotides, amino acids, and lipids as part of generating biomass to support cellular proliferation.^2^ Although in most normal cells increased glycolysis is associated with reduced mitochondrial respiration, increased activity of both these energetic pathways is seen in tumors reflecting the increased metabolic demands of cancer cells.^3^

It has been unclear what role metabolism, and in particular glycolysis, is playing in the pathophysiology of CLL. Previous observations have reported an increase in the mitochondrial mass of CLL cells when compared with healthy B cells, with accompanying increases in mitochondrial respiration, mitochondrial reactive oxygen species (ROS), and oxidative stress.^4–6^ These and other observations regarding increased expression of molecules involved in fatty acid metabolism have led some authors to suggest that glycolysis does not play a key role in CLL cells’ metabolism, with the focus instead placed upon mitochondrial respiration/OXPHOS.^7^ However, other data have suggested that while this interpretation may be correct for quiescent CLL cells in the peripheral blood, CLL cells may alter their metabolism upon entry to the tissues where they proliferate.^8^ Contact with microenvironmental stromal cells has been demonstrated to lead to increased glucose uptake, expression of glucose transporters, and glycolytic enzymes driven by Notch-MYC signaling.^9^ Furthermore, CLL cells have been demonstrated to be primed for the hypoxic conditions that exist within proliferation centers (PCs) in CLL lymph nodes, where they upregulate hypoxia inducible factor 1α (HIF-1α), which also induces glycolysis.^10,11^ There is also some evidence that B-cell receptor (BCR) signaling may be playing a role in CLL metabolism as other authors have shown that genetic or pharmacological inhibition of PI3Kδ results in a reduction in the extracellular acidification rate (ECAR) of CLL cells,^12,13^ whereas BCR-stimulation of healthy B cells can induce glucose uptake.^14^ However, the impact of BCR-stimulation on the metabolism of CLL cells has not been investigated in greater detail.

In this report, we demonstrate increased expression of glycolytic and mitochondrial markers within CLL PCs, with a concomitant increase in the metabolic activity of the CXCR4^low^CD5^high^ fraction of peripheral blood CLL cells that have recently egressed from the lymph node.^15^ We show that anti-IgM stimulation of CLL cells causes them to adopt many features of the “Warburg effect,” including increased glucose uptake, increased lactate production, and induction of glycolytic enzyme expression. Treatment of CLL cells in vitro by inhibitors of BCR-signaling and MYC blocked the anti-IgM-induced changes, and CLL cells from patients on ibrutinib showed strong reduction in basal levels of hexokinase 2 (HK2) with treatment. Interestingly, we identified a group of primary CLL samples with 17p-deletion as being more glycolytic with increased spontaneous lactate production, suggesting a more BCR-independent metabolic phenotype. We conclude that BCR-stimulation causes a switch to aerobic glycolysis and upregulation of respiratory reserve, whereas a subgroup of CLL cells with 17p-deletion manifests these metabolic features spontaneously.

## MATERIALS AND METHODS

### Patients

Peripheral blood mononuclear cells (PBMCs) were obtained from untreated CLL patients from the CLL Research Consortium tissue core (San Diego, USA). Additional PB and lymphoid tissue samples were obtained from Barts Cancer Institute tissue bank, London, United Kingdom (Suppl. Tables S1 and S2). All patients had been consented in accordance with the Declaration of Helsinki, and all studies approved by Ethical Review Boards.

### Immunohistochemistry

Tissue microarrays of triplicate 1-mm diameter cores were prepared from paraffin blocks using a manual tissue arrayer (Beecher Scientific, Estigen, Estonia) as previously described.^16^ “CLL-cell rich” cores with >80% CD79a^+^ cells were used for analysis. Proliferating cells were detected based on their expression of Ki67 (Agilent). The following other antibodies were used: anti-MYC was from Abcam; anti-hexokinase 2 was from Sigma; anti-GLUT3 was from Novus; anti-pyruvate kinase M1, pyruvate kinase M2, and PFKFB3 were from Cell Signaling Technology; anti-NDUFS3, TOMM20, and succinate dehydrogenase complex subunit A (SDHA) were from Abcam. The primary antibody reaction was detected using a peroxidase-labeled system (Super Sensitive Polymer-HRP IHC Detection System; BioGenex, Fremont, USA). Heat induced epitope retrieval (citrate) was used for all antibodies. The slides were scanned with the Pannoramic 250 Flash II system. Immunostaining was quantified by computerized image analysis using the DensitoQuant tool in CaseViewer (3DHistTech, Budapest, Hungary).

### Isolation of CLL cells from PBMCs

CLL cells were negatively isolated using the B-CLL isolation kit from Miltenyi Biotec (Woking, United Kingdom) according to the manufacturer’s instructions. Cell viability was >80% and the proportion of CD19^+^ cells was >90% in all cases.

### BCR-stimulation and inhibitors

BCR-stimulation was evaluated after cells were treated with 20 μg/mL goat F(ab′)^2^ antihuman IgM (Jackson, Cambridge, United Kingdom) or antihuman IgD or antihuman IgG isotype control (Southern Biotechnology, Cambridge, United Kingdom) at 37°C for various times as discussed in the text. The following inhibitors were used: ibrutinib (Janssen, Beerse, Belgium), 2-deoxy-D-glucose (2-DG; Sigma), idelalisib and JQ1 (Selleckchem, Houston, USA). Ibrutinib and idelalisib were used at a concentration of 1 µM, JQ1 was 10 nM. To determine the level of synergy between ibrutinib and 2-DG and their IC50 values, ibrutinib was used at concentrations of 0.01 µM, 0.1 µM, 1 µM, 10 µM, 20 µM, and 50 µM, and 2-DG at concentrations of 0.1 mM, 0.5 mM, 1 mM, 2 mM, 4 mM, 8 mM, and 16 mM, as indicated. Further information on the synergy calculation can be found in the Suppl. Methods.

### Staining for intracellular calcium mobilization

Signaling capacity was measured as percentage intracellular Ca^2+^-mobilization as previously described.^17^ Briefly, 10^7^ PBMC/mL were incubated with 4 μM Fluo3-AM (Invitrogen) and 0.02% (vol/vol) Pluronic F-127 (Sigma) for 30 minutes at 37°C. Cells were resuspended at room temperature, then warmed to 37°C for 5 minutes before acquisition for 35 seconds to obtain the background fluorescence (unstimulated cells). Following addition of antihuman IgM/IgD or control antibodies, data were acquired for 10 minutes, with treatment with 1 μM ionomycin (Sigma) as a positive control. Percent iCa^2+^ mobilization was calculated as [peak (all events) − mean Y (unstimulated cells)/%CD19+ cells] × 100.

### Flow cytometric analysis

Purity of CLL cells was determined by FITC conjugated CD19 staining (Invitrogen; Thermo Fisher Scientific). Where indicated Annexin V (BD Pharmingen, United Kingdom) was used to assess viability. Proliferative and resting CLL fractions were determined by CXCR4 and CD5 staining as described elsewhere.^15^ Briefly, CXCR4 (PE conjugated; Invitrogen, Thermo Fisher Scientific or APC conjugated; Biolegend) and CD5 (FITC and AF647 conjugated; Southern Biotech, Cambridge Bioscience or APC conjugated; Life Technologies) antibodies were used. The fractions were gated as ~5% of each of CXCR4^high^CD5^low^ (resting fraction), CXCR4^low^CD5^high^ (proliferative fraction), and the intermediate between the two above. Ki67-BV421 (Biolegend) was used to assess proliferation. For assessment of glucose uptake by flow cytometry, cells were first preincubated in media without glucose for 45 minutes at 37°C and then 2-(N-(7-Nitrobenz-2-oxa-1,3-diazol-4-yl)Amino)-2-Deoxyglucose (2NBDG, Abcam, United Kingdom) was added at concentration 200 µM and cells were incubated for further 15 minutes at 37°C. This was followed by wash with PBS supplemented with 2% FBS and staining with CXCR4/CD5 antibodies. For assessment of mitochondrial mass, cells were incubated with 100 nM MitoTracker Green (Invitrogen, Thermo Fisher Scientific) for 30 minutes at 37°C followed by wash and staining with CXCR4/CD5 antibodies. For assessment of mitochondrial transmembrane potential, cells were incubated with 20 nM tetramethylrhodamine, methyl ester (TMRM, Invitrogen, Thermo Fisher Scientific) for 30 minutes at 37°C followed by wash and staining with CXCR4/CD5 antibodies. In all measurements except for Ki67 staining, freshly isolated CLL cells were used and viable cells were assessed by DAPI (Sigma) exclusion. Cells for Ki67 staining were first stained with the CXCR4/CD5 antibodies and the fixable viability dye (eFluor 780, eBioscience, United Kingdom) and then fixed in freezer cold 70% Ethanol. All flow cytometry, with the exception of cell sorting, was performed on a BD Fortessa with analysis using FlowJo software (TreeStar, BD). Cell sort was done on BD FACS Aria.

### Glucose uptake and lactic acid assays

Isolated CLL cells were plated at density of 10^7^ per mL in Glucose-free RPMI1640 (Gibco) supplemented with 10% [vol/vol] dialyzed FCS (Gibco), 2.5% [vol/vol] 1M HEPES (Sigma), 1% [vol/vol] Glutamax (Gibco), and 0.1% [vol/vol] 0.05M β-mercaptoethanol (Sigma) with the addition of 4 mmol/L D-glucose (Sigma). Spent media was collected after 2 days, and the residual glucose and lactate concentrations were measured using a glucose colorimetric detection kit (Invitrogen) and lactate assay kit (Sigma) according to the manufacturer’s instructions.

### Respirometry

Oxygen consumption was measured using Oroboros Oxygraph-2k (Oroboros Instruments, Austria). Freshly isolated or 24 hours cultured, as indicated, CLL cells were centrifuged at 300 *g* and resuspended in the media as used for the glucose and lactate assays. After stabilization of the signal, chambers were closed and the stabilized signal recorded as routine respiration. Sequential additions of 0.5 µM Carbonyl cyanide-*p*-trifluoromethoxyphenylhydrazone (FCCP) allowed for assessment of the maximal respiration (electron transfer system capacity). Rotenone at a concentration of 0.5 µM was used to inhibit complex I and 2.5 µM antimycin A to inhibit complex III. Signal recorded after antimycin A addition was subtracted from all recorded values for the evaluation. Reserve respiratory capacity was calculated by subtracting routine respiration from the electron transfer system values. DatLab software (Oroboros, Austria) was used for both the recordings and evaluations.

### Immunoblot analysis

Lysates were mixed with Laemmli buffer and run on precasted tris/glycine gels (Bio-rad Laboratories Ltd) and proteins transferred to PVDF membranes (Bio-rad Laboratories Ltd) using the semidry transfer (Trans Blot Turbo, Bio-rad Laboratories Ltd). Cells were incubated with primary and secondary antibodies and membranes visualized with the ECL substrate (Thermo Scientific). Full description of the methodology can be found in the Suppl. Appendix.

### DNA isolation and sequencing

DNA was isolated using the DNeasy Blood and Tissue kit (Qiagen) according to manufacturer’s instructions and sequencing performed using Ion Ampliseq Cancer Hotspot 50-gene panel. For further details, please see the description in the Suppl. Appendix.

### Statistical analysis

Statistical analyses were performed using Prism Version software (GraphPad, San Diego, USA). ns = not significant; *P* > 0.05; *P < 0.05; **P < 0.01; ***P < 0.001; ****P < 0.0001 in the figures.

## RESULTS

### Increased expression of glycolytic and mitochondrial markers within proliferation centers in CLL lymph nodes

Proliferation centers are a key feature of CLL lymph nodes, where they are thought to be important sites of B-cell receptor (BCR) signaling and cellular proliferation.^18,19^ Surface IgM stimulation is known to induce MYC expression in CLL cells in vitro, which correlates with observations regarding increased MYC expression within CLL PCs.^19,20^ Despite the importance of these sites for CLL progression, and the role of MYC as a master regulator of metabolism,^21^ little is known about the metabolic events that occur within CLL PCs.

**Figure 1.**
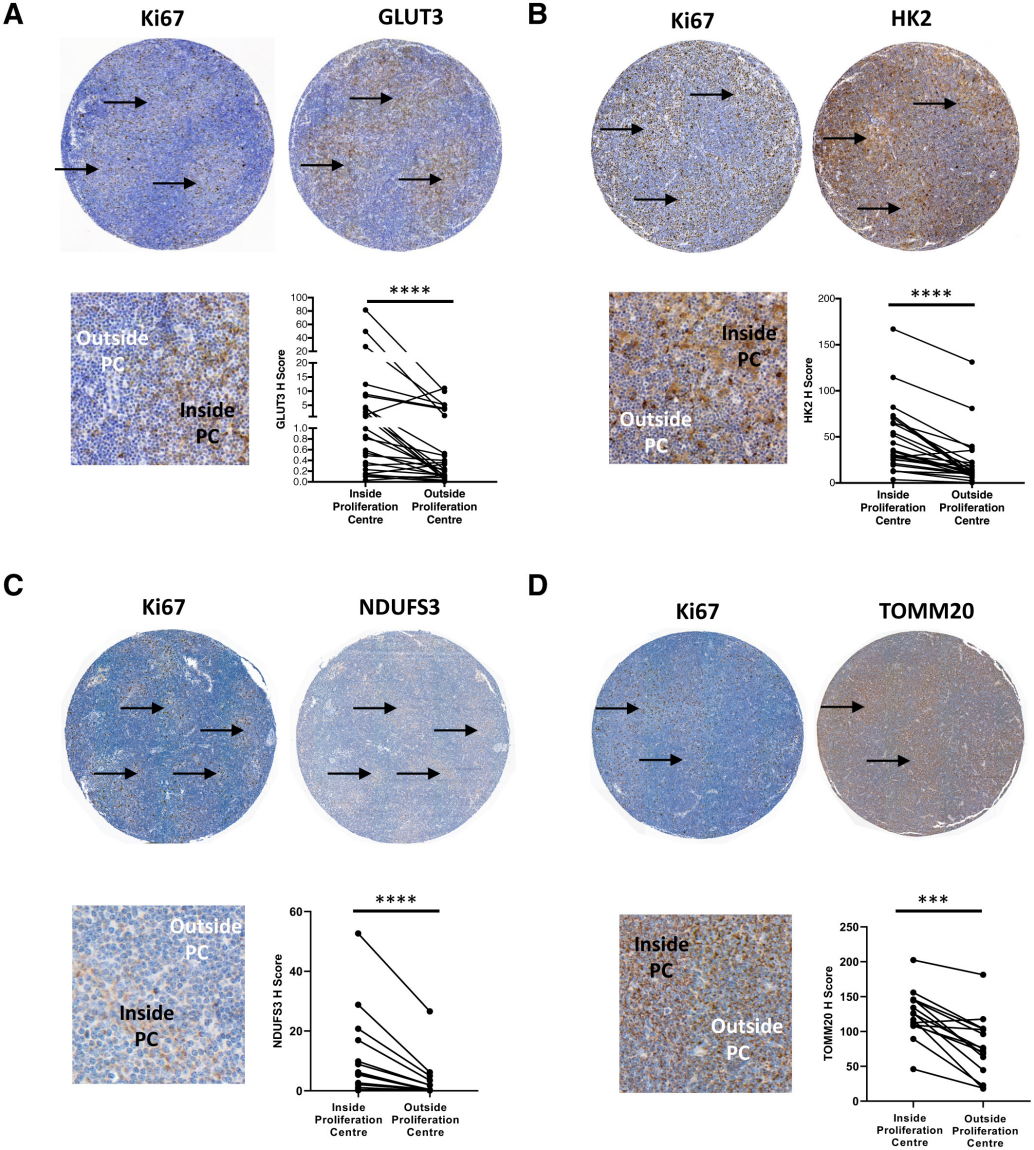
**Increased expression of glycolytic and mitochondrial markers within proliferation centers in CLL lymph nodes.** The expression of GLUT3 (A), HK2 (B), NDUFS3 (C), and TOMM20 (D) was assessed in lymph node CLL cells by immunohistochemistry. Representative images of whole cores are shown. Detailed images show higher expression of Ki67 (A, B, C, D), GLUT3 (A), HK2 (B), NDUFS3 (C), and TOMM20 (D) in CLL cells within proliferation centers (arrowed). n = 26 for GLUT3 and HK2, n = 15 for NDUFS3 and TOMM20. CLL = chronic lymphocytic leukemia.

We examined the expression of a panel of metabolic enzymes and transporters in tissue microarrays composed of lymphoid tissue biopsies from CLL patients. PCs were identified based on their morphology and the presence of an increased proportion of Ki67^+^ cells (Figure 1). Consistent with previous reports we observed an increase in the expression of MYC within PCs (Suppl. Figure S1A). The expression of glycolytic enzymes glucose transporter 3 (GLUT3), hexokinase 2 (HK2), and phosphofructo-2-kinase/fructose-2,6-biphosphatase 3 (PFKFB3) was increased in cells within PCs compared with those outside PCs (Figure 1A and B; Suppl. Figure S1B). Similarly, we found increased expression of NADH:ubiquinone oxidoreductase core subunit S3 (NDUFS3) (Figure 1C) and succinate dehydrogenase complex subunit A (SDHA) (Suppl. Figure S1C), subunits of mitochondrial respiratory complexes I and II, and of mitochondrial outer membrane transporter translocase of outer mitochondrial membrane 20 (TOMM20) (Figure 1D). Quantification of the intensity of staining in lymphoid tissue biopsies confirmed that cells within PCs had consistently higher expression of all metabolic enzymes tested in comparison to those outside PCs (*P* < 0.0001 for GLUT3, HK2, and NDUFS3; *P* = 0.0001 for TOMM20; Figure 1A–D and Suppl. Figures S1B and C; *P* = 0.0001).

Pyruvate kinase (PKM) catalyzes the dephosphorylation of phosphoenolpyruvate to pyruvate representing the final step in glycolysis. The embryonic pyruvate kinase isoform, PKM2, is almost universally re-expressed in cancer and is the predominant isoform of pyruvate kinase expressed in normal hematopoietic and leukemic cells.^11,22^ Interestingly, we observed that PKM2 was expressed at very high levels within CLL lymph nodes, where it was not confined to PCs, whereas PKM1 was expressed at low levels. This was in marked contrast to cardiac muscle, where the reverse pattern was observed (Suppl. Figure S1D). However, when the effect of disease progression on the relative levels of PKM2 and PKM1 in circulating CLL cells was assessed, there was a decrease in PKM1 in cells from patients with progressive disease compared with cells from patients with indolent disease while PKM2 levels were maintained resulting in an increase in the PKM2:PKM1 ratio with disease progression (Suppl. Figure S1E and F; *P* = 0.0027). Further, although no change in the expression of GLUT3 and PFKFB3 was detected between the CLL cells in patients with indolent or progressive disease, we found increased levels of HK2 in cells from patients with CLL progression (Suppl. Figure S1G; *P* = 0.0175). In summary, these findings provide strong in vivo evidence of increased metabolic activity of CLL cells within the PCs in lymphoid tissues.

### Circulating CXCR4^low^CD5^high^ CLL cells exhibit increased glycolysis and mitochondrial respiration

**Figure 2.**
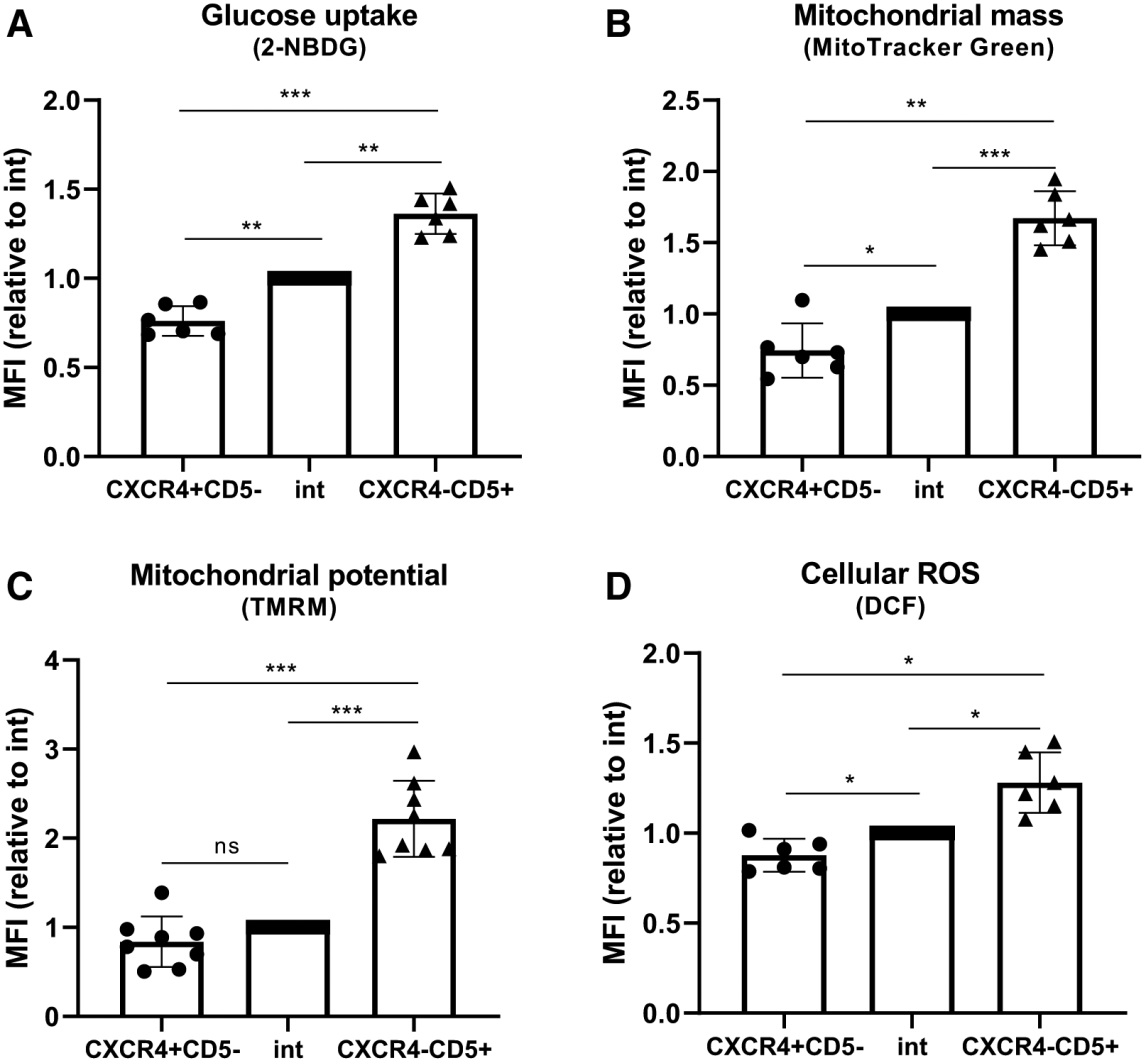
**Circulating CXCR4^low^CD5^high^ CLL cells exhibit increased metabolic activity.** Primary CLL cells were incubated with 2NBDG (A), MitoTracker Green (B), TMRM (C), and DCF (D), labeled with CXCR4 and CD5 antibodies and analyzed by flow cytometry. CXCR4^high^CD5^low^, intermediate and CXCR4^low^CD5^high^ fractions were gated as ~5% of the whole population. The MFI of the metabolic markers is shown relative to intermediate fraction which was set 1. Results in (A, B, D) represent data obtained from 6 different CLL samples in two independent experiments, (C) 8 different CLL samples in 2 independent experiments. Graphs represent mean with standard deviation. CLL = chronic lymphocytic leukemia; MFI = median fluorescent intensity; TMRM = tetramethylrhodamine, methyl ester; DCF = dichlorodihydrofluorescin.

In contrast to CLL cells in PCs, circulating CLL cells in the peripheral blood are known to be relatively quiescent. Despite this, previous reports have identified a CXCR4^low^CD5^high^ fraction of circulating CLL cells that have increased capacity for survival and proliferation.^15^ This population has been suggested to comprise cells that have recently divided within the lymph node and emigrated into the peripheral blood. In light of our initial findings, we hypothesized that this proliferative fraction of circulating CLL cells should therefore have increased metabolic activity when compared with the CXCR4^high^CD5^low^ and CXCR4^int^CD5^int^ fractions, as previously defined.^15^ Consistent with previous reports we were able to identify three fractions based on CD5 and CXCR4 expression (Suppl. Figure S2A), with the CXCR4^low^CD5^high^ fraction having the highest proportion of Ki67^+^ cycling CLL cells (Suppl. Figure S2B). The uptake of fluorescently labeled glucose (2-deoxy-2-[(7-nitro-2,1,3-benzoxadiazol-4-yl)amino]-D-glucose; 2NBDG) in the three fractions was assessed. Notably, 2NBDG uptake increased stepwise with the lowest glucose uptake being detected in the CXCR4^high^CD5^low^ fraction and the highest in the proliferative CXCR4^low^CD5^high^ fraction (Figure 2A). Mitochondrial mass, membrane potential, respiratory capacity, and levels of ROS were also assessed in these three fractions. Importantly, the CXCR4^low^CD5^high^ fraction had significantly higher mitochondrial mass and higher mitochondrial membrane potential compared with the other fractions, which correlated with higher respiratory capacity and ROS levels (Figure 2B–D; Suppl. Figure S2C). These results confirm that proliferating CLL cells significantly increase glycolysis and oxidative phosphorylation, consistent with the changes in expression seen within CLL PCs.

### Anti-IgM stimulation of CLL cells induces glucose uptake and expression of glycolytic enzymes and transporters

**Figure 3.**
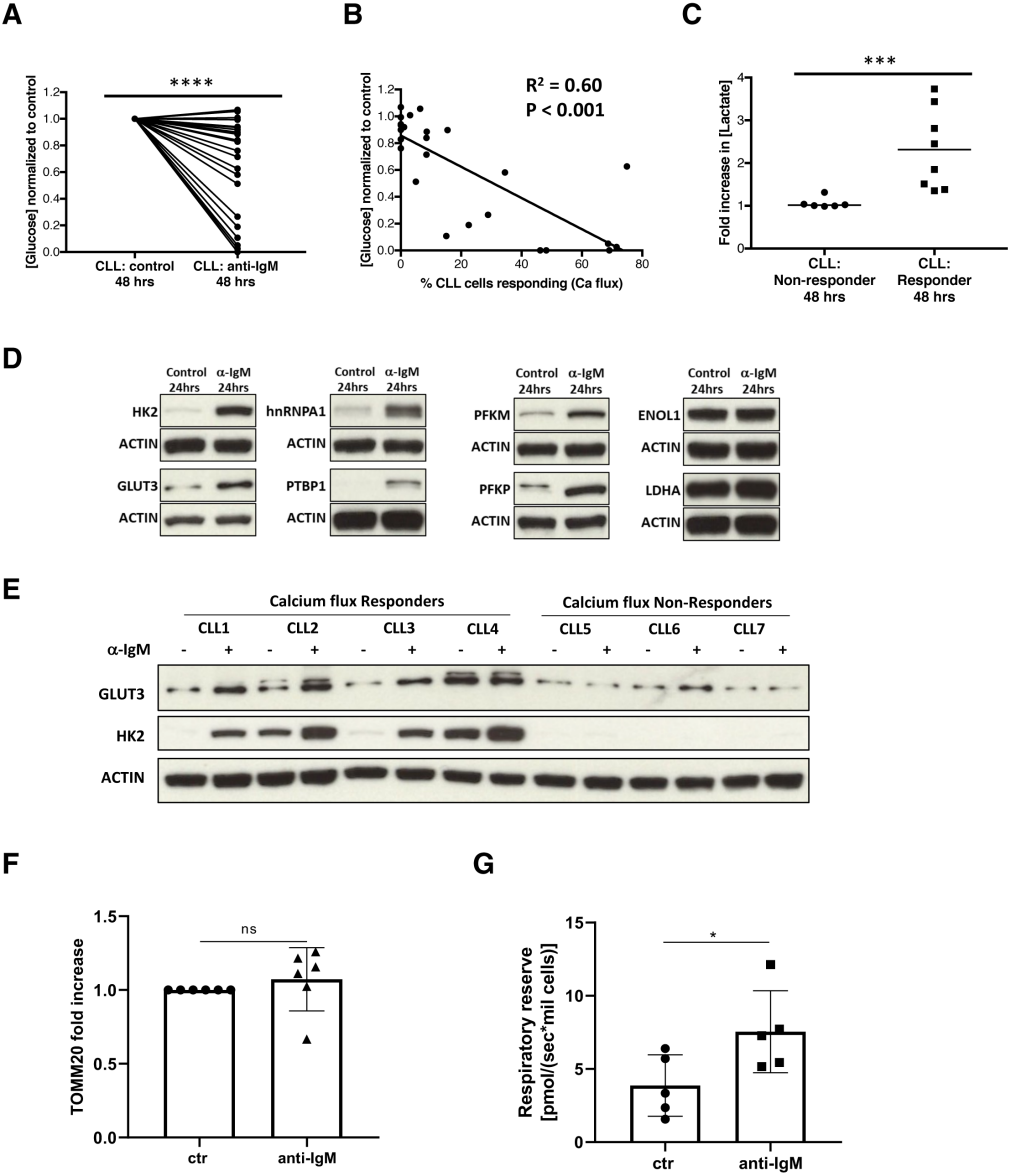
**Increased glucose uptake after anti-IgM stimulation of CLL cells.** CLL cells were cultured for 48 hours with either anti-IgM stimulation or control and the residual concentration of glucose ([glucose]^residual^) in the media was assessed by colorimetric assay. (A) The [glucose]^residual^ was lower when CLL cells (n = 26) were stimulated with anti-IgM than when stimulated with isotype control indicating greater glucose uptake with anti-IgM stimulation. (B) The [glucose]^residual^ was correlated with the percentage of CLL cells that exhibited intracellular calcium flux after IgM stimulation (n = 26). There was a significant correlation between [glucose]^residual^ and the proportion of CLL cells exhibiting calcium flux, with samples where the majority of cells mobilized calcium also showing the highest degree of glucose uptake. (C) The concentration of lactate ([lactate]) in the media after 48 hours culture was assessed by colorimetric assay. Samples that exhibited calcium flux after anti-IgM stimulation and high glucose uptake (n = 8) had a significant increase in the [lactate] in the media at the end of the culture period compared with nonresponders (n = 6). (D) CLL cells were stimulated by anti-IgM or isotype control for 24 hours and the expression of a panel of glycolytic enzymes was assessed by immunoblot. Representative blots of HK2, GLUT3, PFKM, PFKP, hnRNPA1, PTBP, ENOL-1, and LDHA after anti-IgM stimulation or isotype control. (E) Representative blots comparing the impact of anti-IgM stimulation on HK2 and GLUT3 expression in calcium flux responders and nonresponders. (F) There was no increase in TOMM20 expression in CLL cells stimulated with anti-IgM or isotype control (n = 6). (G) 24 hours anti-IgM treatment resulted in increased respiratory reserve (n = 5). CLL = chronic lymphocytic leukemia; LDHA = lactate dehydrogenase.

We proceeded to interrogate the potential factors that were inducing these metabolic alterations by exposing peripheral blood CLL cells to stimuli typically found in the microenvironment of the CLL lymph node. B-cell receptor (BCR) signaling plays a crucial role in the pathogenesis of CLL as evidenced by the prognostic importance of *IGHV*-mutation status, the presence of “stereotyped” BCRs and the clinical efficacy of drugs such as ibrutinib.^23–25^ Although recent reports have suggested that BCR-signaling regulates CLL-cell metabolism these investigators used ECAR as an indirect measure of glycolysis and did not investigate the impact of BCR-stimulation on glucose uptake.^13^ Isolated CLL cells were stimulated with soluble anti-IgM, anti-IgD, or control antibody and cultured for 48 hours before the residual glucose in the media was assessed. Anti-IgM stimulation resulted in a significant reduction in residual glucose concentration indicating glucose uptake (*P* < 0.0001; Figure 3A). However, the response to anti-IgM was very heterogenous with some samples showing modest glucose uptake, while other samples consumed all of the glucose in the medium. It is known that there is a wide variation in the ability of CLL cells to mobilize intracellular calcium and phosphorylate Syk in response to anti-IgM stimulation.^26,27^ In light of this, the signaling capacity of CLL cells measured by calcium flux was correlated with glucose uptake. There was a significant correlation between calcium flux and glucose uptake, with samples with high calcium flux also showing the highest degree of glycolytic activity (*P* < 0.001; Figure 3B). The concentration of lactate in the media after 48 hours culture was also compared between calcium flux responders and nonresponders. This dichotomy was defined in accordance with previous reports using calcium flux in >5% of cells as the cutoff for a “responder.”^26^ Samples that exhibited a calcium flux response to anti-IgM stimulation had a significant increase in lactate concentration compared to nonresponders (*P* = 0.0007; Figure 3C). In contrast anti-IgD stimulation had a minimal effect on glucose uptake by CLL cells even in cases that responded to anti-IgM stimulation (Suppl. Figure S3A).

We then investigated the impact of BCR-stimulation on a variety of glycolytic transporters and enzymes. We focused on samples exhibiting the most robust calcium flux and glucose uptake response to anti-IgM for these and subsequent experiments. We and others have previously demonstrated that MYC is induced by BCR-signaling in CLL cells, with the ability of anti-IgM stimulation to increase MYC expression being correlated with calcium flux.^19,28,29^ MYC is known to enhance glucose uptake by upregulating glucose transporters and by transcriptional regulation of many glycolytic genes, in particular HK2, type-1 phosphofructokinases (PFKM and PFKP), enolase (ENOL-1), and lactate dehydrogenase (LDHA).^30–32^ In addition, MYC induces expression of the heterogenous nuclear ribonuclearproteins (hnRNPs) A1, A2/B1, and PTBP1 to regulate pyruvate kinase mRNA splicing.^33^ Although levels of HK2, GLUT3, hnRNPA1, PTBP1, PFKM, and PFKP were all low in resting CLL cells, they were significantly induced by anti-IgM stimulation at 24 hours (Figure 3D; Suppl. Figure S3B). There was a correlation with signaling capacity as calcium flux responders exhibited higher fold-increases in GLUT3 and HK2 expression than nonresponders (Figure 3E; Suppl. Figure S3C). Interestingly, the other MYC targets ENOL-1 and LDHA were constitutively expressed with LDHA only marginally increasing after anti-IgM stimulation (Figure 3D; Suppl. Figure S3B). Other glycolytic enzymes including hexokinase 1 and liver-phosphofructokinase were also constitutively expressed and did not increase further after anti-IgM stimulation (Suppl. Figure S3B). Anti-IgD stimulation had a similar but more modest effect on the expression of hnRNPA1 and GLUT3 with no effect on HK2 (Suppl. Figure S3D). Although there was no difference in mitochondrial mass after anti-IgM stimulation (Figure 3F), this did lead to an increase in CLL cell respiratory reserve (Figure 3G).

### Inhibition of BCR-signaling and MYC transcription metabolically re-programs CLL cells in vitro and in vivo

**Figure 4.**
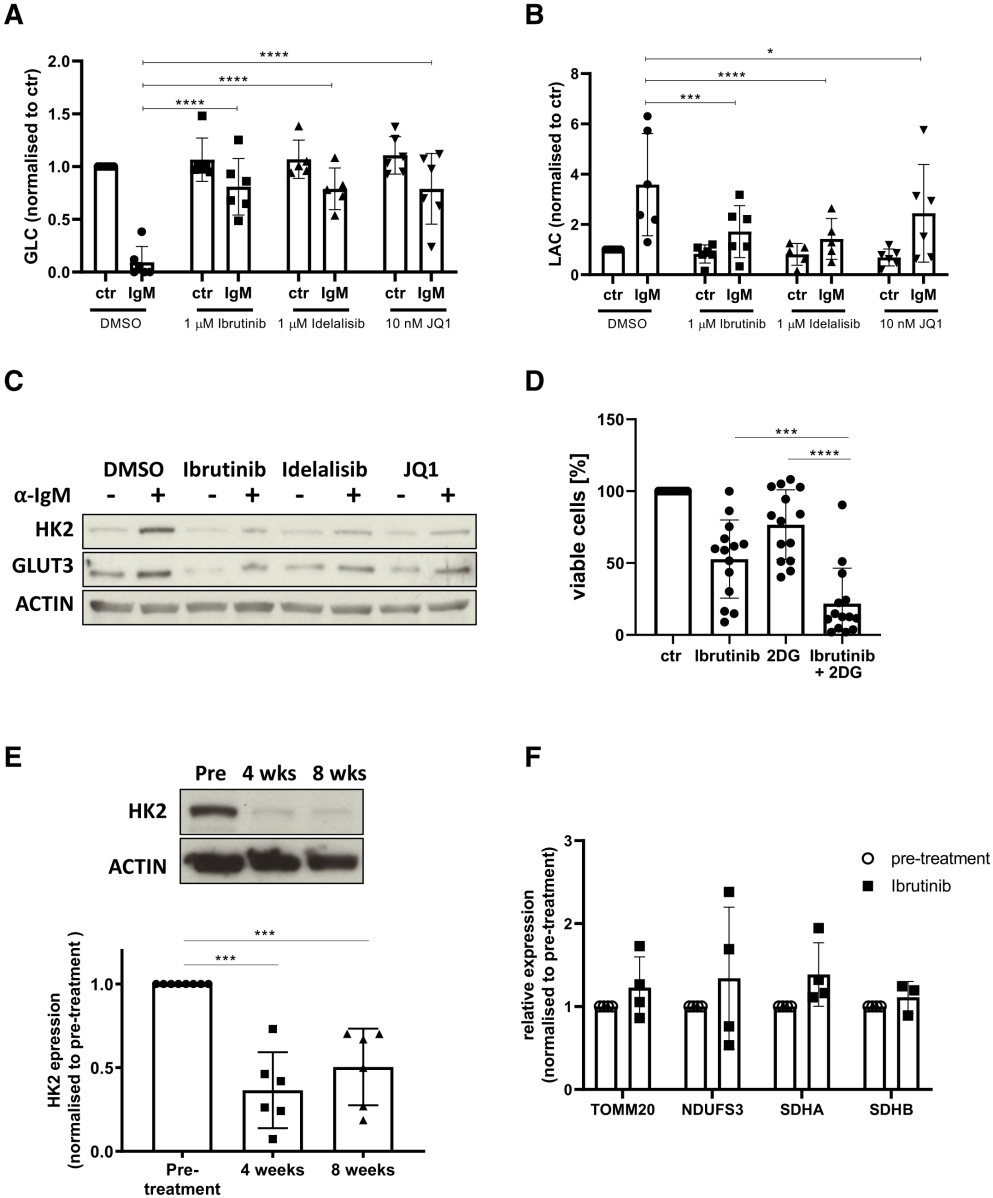
**Inhibition of BCR-signaling and MYC transcription metabolically reprogram CLL cells in vitro and in vivo.** (A, B) CLL cells (n = 6) were cultured for 48 hours in the presence of the inhibitors or vehicle control (DMSO) and the residual concentration of glucose (A) and lactate (B) in the media was assessed. There was a significant reduction in glucose and increase in lactate with anti-IgM stimulation, which was inhibited by ibrutinib, idelalisib, and JQ1. (C) Representative blot demonstrating the impact of the inhibitors on the induction of HK2 and GLUT3 after anti-IgM stimulation for 24 hours. The induction of HK2 (n = 7) and GLUT3 (n = 7) was also inhibited by ibrutinib, idelalisib, and JQ1. (D) CLL cells (n = 14) were cultured for 48 hours in the presence of 10 µM ibrutinib (or vehicle control—DMSO) and 1 mM 2-deoxyglucose (2-DG) as indicated and apoptosis was assessed by Annexin V binding. Addition of 2-DG resulted in increased sensitivity to ibrutinib. (E) The impact of ibrutinib on the expression of HK2 in peripheral blood CLL cells was assessed in previously untreated patients initiating treatment with single-agent ibrutinib. Representative blot of HK2 showing a reduction in expression at 4 and 8 weeks of treatment in vivo. The expression of HK2 decreased significantly in all cases after 4 weeks (n = 6) and 8 weeks (n = 6) of treatment. (F) In contrast, the expression of TOMM20 (n = 4), NDUFS3 (n = 4), SDHA (n = 4), and SDHB (n = 3) did not change after ibrutinib treatment. BCR = B-cell receptor; CLL = chronic lymphocytic leukemia; DMSO = dimethyl sulfoxide; SDHA, succinate dehydrogenase complex subunit A; SDHB, succinate dehydrogenase complex subunit B.

A variety of agents developed to inhibit BCR-signaling have shown significant clinical activity in CLL including the Bruton tyrosine kinase (BTK) inhibitor, ibrutinib, and the phosphoinositide 3-kinase inhibitor, idelalisib.^18,23,34^ In light of their known clinical efficacy the effect of these agents on glucose uptake and enzyme induction was assessed. MYC was also targeted by use of the bromodomain inhibitor, JQ1, due to its efficacy in other models of hematological malignancies.^35^ The increase in glucose uptake induced by anti-IgM stimulation was blocked by ibrutinib, idelalisib, and JQ1 (Figure 4A). Consistent with this, all three drugs also inhibited the anti-IgM induced lactate production (Figure 4B) and the increase in HK2 and GLUT3 (Figure 4C). We did not observe any significant impact of these drugs on CLL-cell viability or number after 48-hour culture at the concentrations used (ibrutinib and idelalisib:1μM; JQ1:100 nM; Suppl. Figure S4A and B). In light of the above observations, we hypothesized that inhibition of glycolysis with 2-DG (IC50 = 4 mM; Suppl. Figure S4C) would sensitize CLL cells to ibrutinib (IC50 = 7.3 µM; Suppl. Figure S4C). We determined the interaction between the two drugs as synergistic (defined as synergy score >10) with an overall synergy score of 17.5 (Suppl. Figure S4D), with the highest synergy score (44.17) observed with 10 µM ibrutinib combined with 1 mM 2-DG. This combination of drugs led to a significant increase in apoptosis, compared with when the cells were treated with each drug alone (Figure 4D). We also investigated the impact of ibrutinib on the basal expression of GLUT3 and HK2 in a cohort of previously untreated patients initiating treatment with single-agent ibrutinib (420 mg daily). PB samples were drawn immediately before starting ibrutinib and then subsequently after 4 and 8 weeks of treatment. Expression of HK2 was significantly reduced at both 4 and 8 weeks compared with pretreatment in all cases (Figure 4E), whereas the impact on GLUT3 expression was more heterogenous (Suppl. Figure S4E). There was no consistent difference in mitochondrial mass when patients were treated with ibrutinib (Figure 4F).

### 17p-CLL samples are more glycolytic and show increased spontaneous lactate production

**Figure 5.**
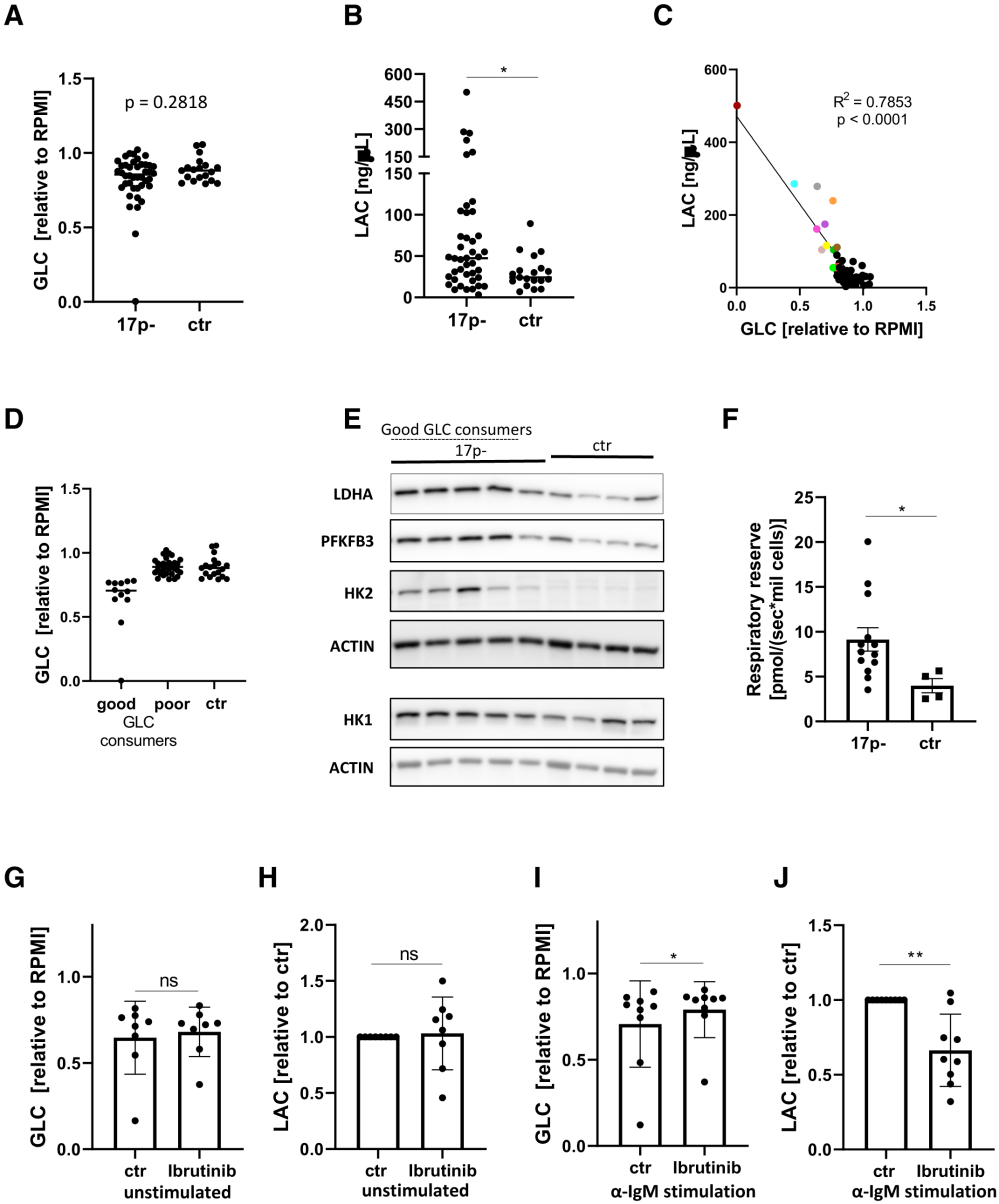
**Metabolic alterations in samples with 17p-.** 17p- (n = 42) and control (non-17p-; n = 19) CLL cells were cultured for 48 hours and the residual concentration of glucose (A) and lactate (B) in the media was assessed. (C) There was a correlation between the spontaneous glucose consumption and lactate production in CLL cells (data from (A) and (B)). CLL cells with the highest glucose consumption (n = 12) are highlighted in colors and respresented as a distinct group “good glucose consumers” in (D). (E) Representative immunoblots of 17p- and control cells show strongly increased expression of HK2, PFKFB3, and LDHA in 17p- cases, particularly in the samples with highest glucose consumption. (F) The respiratory reserve of CLL cells was assessed by treating CLL cells with FCCP and measuring their oxygen consumption. 17p- CLL (n = 13) cells had significantly higher respiratory capacity than non-17p- CLL cells (n = 4). (G–J) 17p-CLL cells were treated with 1µM ibrutinib (or vehicle control—DMSO) for 48 hours in the unstimulated (G, H; n = 8) or anti-IgM stimulated (I, J; n = 9) condition and the spent media was assessed for the residual glucose (G, I) and lactate levels (H, J). CLL = chronic lymphocytic leukemia.

While performing these experiments, we observed that several samples exhibited increased aerobic glycolysis in the unstimulated state, and that these cases invariably carried 17p-deletions (17p-). We compared the data on glucose consumption and lactate production between 42 17p- and 19 control CLL samples (no 17p-). Although there was no overall change in the spontaneous glucose consumption between the groups (17p- median = 0.8556; control median = 0.8813; *P* = 0.2818), we did observe a significant increase in extracellular lactate in the media of 17p-CLL cultures (Figure 5A and B). Several samples from the 17p-group manifested highly increased glucose consumption, which correlated with increased lactate excretion (Figure 5C). We dubbed this subgroup “good glucose consumers” and proceeded to investigate these further (Figure 5D). Our observations from these functional studies were supported by increased expression of the glycolytic enzymes HK2, PFKFB3, and LDHA found in the unstimulated state in the good glucose consumer subgroup (Figure 5E). Despite there being no increase in the expression of mitochondrial markers tested (Suppl. Figure S5A), there was also an increase in the respiratory reserve of 17p- CLL cells (Figure 5F). There was no correlation between the percentage of cells carrying 17p- and spontaneous glucose consumption or lactate production (Suppl. Figure S5B).

We hypothesized that 2-DG treatment of the spontaneously glycolytic 17p- samples might sensitize them to ibrutinib. Surprisingly, we observed that this group was more resistant to this combination (Suppl. Figure S5C). This observation may be explained by our finding that these samples had an increased respiratory reserve, which would allow for greater metabolic “flexibility” (eg, mitochondrial oxidation) when glycolysis is inhibited (Suppl. Figure S5D). We then investigated whether ibrutinib would inhibit glycolysis in the 17p- CLL samples. Although ibrutinib had no effect on spontaneous glucose consumption and lactate production, it did reduce glycolysis induced by anti-IgM stimulation (Figure 5G–J). This result suggests that while the increased basal glycolysis seen in 17p- CLL cells is BTK-independent, 17p- CLL cells do still exhibit a metabolic switch to glycolysis upon anti-IgM stimulation that is sensitive to ibrutinib.

It has been well characterized that 17p- is often accompanied by a *TP53* mutation in the other allele.^36^ Interestingly, some of these mutations are known to be gain of function (GOF) mutations and have been associated with changes in metabolism, in particular increased glycolysis.^37^ In light of this, we hypothesized that the functional behavior of the good consumer subgroup may be due the presence of these GOF *TP53* mutations. However, *TP53* gene sequencing of 34 CLL samples (12 [all] × 17p- good consumers; 14 × 17p- poor consumers; 8× non-17p- control samples) did not show any enrichment for GOF *TP53* mutations within this subgroup (Suppl. Table S3). We did not observe any pattern in mutation types with both good and poor glucose consumers having missense *TP53* mutations in the other allele. Furthermore, we could not detect a *TP53* mutation in two of the good glucose consumers; we also found one frame-shift mutation in this group. Nevertheless, we cannot exclude the possibility that some of the detected missense mutations found in the good glucose consumers could be a yet unrecognized GOF mutation contributing to the increased spontaneously glycolytic phenotype.

As we observed increased metabolic activity in the CXCR4^low^CD5^high^ proliferative fraction of circulating CLL cells, we hypothesized that alterations in trafficking may contribute to the spontaneous glycolytic phenotype in 17p- samples. However, there were no clear and consistent differences in CD5/CXCR4 expression between the subgroups (Suppl. Figure S5E). Next, we considered the possibility that the reason for the observed spontaneous glycolysis in the subgroup of 17p- samples could be due to increased constitutive expression of MYC or HIF transcription factors, which are known modulators of metabolism and glycolysis, including in CLL. Despite a recent report showing overexpression of HIF-1α in *TP53*-disrupted patients, we did not observe an increase in HIF-1α expression (or a decrease in VHL ubiquitin ligase expression) in the good consumer subgroup (Suppl. Figure S5F).^38^ In contrast, basal MYC expression was strongly increased in several of the good consumers following expression pattern similar to that of LDHA and HK2 in some of the good glucose consumers (Suppl. Figure S5G). Taken together these data provide evidence that 17p- is associated with alterations in the metabolism of CLL cells.

## DISCUSSION

In this study, we investigated the metabolic alterations that occur in CLL cells when they are activated in vivo and in vitro. By studying the metabolic differences that occur within CLL-cell populations from the same patient, we have shown that CLL cells in PCs express higher levels of glycolytic enzymes and transporters and mitochondrial markers, which correlates with higher glucose uptake and oxidative phosphorylation in CXCR4^low^CD5^high^ cells that have recently left the lymph node microenvironment. Anti-IgM-activation of CLL cells causes them to take up more glucose and increase their expression of glucose transporters and key glycolytic enzymes such as HK2, with the extent of these metabolic alterations reflected by levels of initial calcium flux. Taken together, it seems likely that while CLL cells are relatively quiescent in the peripheral blood, BCR-signaling in PCs in LNs promotes an upregulation of glycolysis and oxidative phosphorylation to support cellular proliferation and drive the disease.

It is likely that BCR-signaling is not the only pathway to promote the switch to glycolysis upon entry to the tissues. Previous reports have shown that CLL-cell interactions with stromal cells promote a MYC-induced glycolytic switch mediated by NOTCH signaling.^9^ Other investigators have shown that hypoxia within CLL lymph nodes may also be playing a role. HIF-1α is known to induce glycolysis as part of maintaining cellular energy production in the absence of oxygen. HIF-1α is expressed with CLL PCs and promotes glycolysis in CLL cells that appear “primed” for hypoxic conditions.^10,11^ Therefore, it seems likely that the combination of antigen, interactions with stromal cells and hypoxia all combine to promote a switch to glycolysis within CLL PCs to support CLL-cell proliferation. An intriguing aspect of the clinical efficacy of BCR-signaling inhibitors such as ibrutinib is that they affect chemokine and integrin signaling leading to a redistribution of CLL cells from the lymph nodes into the peripheral blood.^23,39,40^ Therefore, while drugs such as ibrutinib metabolically reprogram CLL cells directly by inhibiting BCR-signaling, the movement of CLL cells out of the lymph nodes will also inhibit glycolysis by reducing interactions with stromal cells and exposure to hypoxia in the CLL nodal microenvironment.

Although the majority of CLL cells were relatively metabolically quiescent in the absence of stimulation, we did observe that 17p- CLL cells exhibited increased basal metabolism with increased lactate production and respiratory reserve. Within this group, there was a subgroup of cases (~30% of 17p- cases) where the cells had very pronounced increases in resting glucose uptake associated with higher resting expression of HK2, PFKFB3, LDHA, and MYC. This suggests that the presence of *TP53* abnormalities enables CLL cells to partially bypass the requirement for BCR-signaling to induce metabolic reprogramming, with these cells becoming less dependent on microenvironment stimuli. It should be noted, however, that p53 itself suppresses glycolysis and oncogenic signaling, so p53 deficiency might be the reason behind the increased glycolysis and MYC in some of the 17p- cells.^21,41^

Our data are particularly interesting in light of a previous report, which correlated^18^ fluorodeoxyglucose positron emission tomography (FDG/PET) data, histological diagnosis, clinical characteristics, and survival in patients with CLL. The CLL samples were divided into “histologically indolent” CLL or “histologically aggressive” CLL with the latter having increased large cells, large confluent PCs, or higher expression of Ki67, comparable to “accelerated” CLL.^42,43^ Notably, the histologically aggressive cases had an increased maximum standardized uptake value (SUV_max_) on FDG/PET imaging, reflecting higher glucose uptake in vivo. They also had a significantly poorer prognosis comparable to patients with Richter syndrome (RS). These observations would be predicted by our own findings, as several studies have shown that increased expression of GLUT3 and HK2 correlates with higher SUV_max_ in other lymphomas.^44–47^ Taken together, this suggests that glycolysis is a crucial process in CLL biology with increased glycolytic capacity promoting a poorer prognosis in this disease. Our work also has important implications for the understanding of the biology of transformation to RS. Our observations regarding increased resting metabolic activity of 17p- CLL cells, with particularly high glucose uptake in a subgroup with high MYC expression, is intriguing in view of the known importance of lesions in *MYC* and *TP53* in RS.^48^ Furthermore, recent observations have suggested that FDG-PET may be less sensitive and specific for the diagnosis of RS, when it is used in patients who have been treated with BCR-signaling inhibitors such as ibrutinib.^49^ As we demonstrate here that BCR-signaling inhibition blocks anti-IgM induced alterations in metabolism, we hypothesize that high-grade transformation in this context must be accompanied by genetic event such as 17p- that bypass the “metabolic block” caused by drugs such as ibrutinib and idelalisib. Understanding this interaction between metabolism, genetics, and BCR-signaling will allow for the development for novel therapeutic approaches for the treatment of CLL and RS.^50^

## ACKNOWLEDGMENTS

The authors are very grateful to all of the patients from both the United States and United Kingdom who generously donated their blood and tissue to the tissue banks. We thank Professor Karen H. Vousden for her support and advice. We thank Paul Grevitt for kindly providing the VHL antibody.

## AUTHOR CONTRIBUTIONS

KK and AJC designed and performed the experiments, analyzed and interpreted data, and wrote the article. AD designed and performed experiments, analyzed, and interpreted data. LZR and TJK provided the samples and edited the article. JGG designed experiments and edited the article, and JCR designed and performed the experiments, analyzed and interpreted the data, wrote and edited the article, and supervised the study. All authors approved the final submission.

## SOURCES OF FUNDING

This work was supported by grant 110020/Z/15/Z from the Wellcome Trust (JCR), grant KKL128 from the Kay Kendall Leukaemia Fund (JCR), Cancer Research UK Centre Grant (C355/A25137), and Core Service Grant at Barts Cancer Institute (Core Award C16420/A18066) and by funding from the National Cancer Institute (P01 CA95426; JGG, LZR, and TJK).

## DISCLOSURES

JGG has received research funding from Celgene, Janssen, and Acerta and honoraria from Abbvie, Acerta, Celgene, Gilead, Janssen, Novartis, Pharmacyclics, and Roche. All the other authors have no conflicts of interest to disclose.

## Supplementary Material


