# Supplementary material for: B-cell Receptor Signaling Induced Metabolic Alterations in Chronic Lymphocytic Leukemia Can Be Partially Bypassed by TP53 Abnormalities
Source: Hemasphere. 2022 May 13;6(6):e722. doi: 10.1097/HS9.0000000000000722 (PMC9208879; doi:10.1097/HS9.0000000000000722)
Supplement: Supplementary file 1 [file hs9-6-e722-s001.pdf]

Supplementary data

Title: B-cell receptor signaling induced metabolic alterations in chronic lymphocytic leukemia can be partially bypassed by *TP53* abnormalities

Supplementary Methods

Immunoblot analysis

Cells were lysed on ice for 30 minutes using lysis buffer (1% [vol/vol] Nonidet P-40, 20mM Tris-HCl, pH 8.0, 150mM NaCl, and 5mM EDTA with protease inhibitors (Roche diagnostics, Burgess Hill, UK) and phosphatase inhibitors (sodium fluoride and sodium orthovanadate (Sigma)). The lysate was cleared by centrifugation at 16 000g. Alternatively, the Nuclear Extract Kit (Active Motif, Cambridge Bioscience) was used to obtain the lysates. Lysates were mixed with Laemmli buffer and run on pre-casted Tris/Glycine gels (Bio-rad Laboratories Ltd) and proteins transferred to PVDF membranes (Bio-rad Laboratories Ltd) using the semi-dry transfer (Trans Blot Turbo, Bio-rad Laboratories Ltd). The following antibodies were used: anti-GLUT1, GLUT4, hexokinase 1, hexokinase 2, platelet phosphofructokinase, enolase 1, pyruvate kinase M1, pyruvate kinase M2, lactate dehydrogenase A, 6-phosphofructo-2-kinase/fructose-2,6-biphosphatase 3, hnRNPA1, hnRNPA2/B1, PTBP1, myc and secondary HRP-conjugated antibodies were all from Cell Signaling Technology (London, UK); anti-GLUT3, NDUFS3, TOMM20, SDHA and SDHB were from Abcam (Cambridge, UK); muscle and liver phosphofructokinase were from Novus (Colorado, USA); anti-actin-HRP was from Santa Cruz (Dallas, USA); anti-HIF1α and VHL were from BD Transduction Laboratories (BD, UK). Membrane bound antibodies were visualised with the ECL substrate (Thermo Scientific). All values were normalized to the actin or Hsc70 loading controls, and relative fold-change was calculated with the isotype control antibody treated cells or patient’s samples before treatment taken as 100% of expression.

Synergy evaluation

For the evaluation of potential synergy between ibrutinib and 2-deoxy-glucose (2-DG) we used 6x6 matrix of 5 different concentrations of ibrutinib (0.01 µM, 0.1 µM, 1 µM, 10 µM, 20 µM) and 2-DG (0.1 mM, 0.5 mM, 1 mM, 2 mM, 4 mM). The impact of these

combinations on apoptosis was assessed by flow cytometry after Annexin V staining. The expected drug combination responses were calculated based on the Zero interaction potency (ZIP) reference model using SynergyFinder.[1] The formulation of the model can be found at https://www.ncbi.nlm.nih.gov/pmc/articles/PMC4759128/. The ZIP model takes the advantages of both the Loewe additivity and the Bliss independence models, aiming at a systematic assessment of various types of drug interactions patterns that may arise in a high-throughput drug combination screening. Deviations between observed and expected responses with positive and negative values denote synergy and antagonism respectively.

If synergy score is:

- Less than -10: the interaction between two drugs is likely to be antagonistic;
- From -10 to 10: the interaction between two drugs is likely to be additive;
- Larger than 10: the interaction between two drugs is likely to be synergistic.

Sequencing

Mutation detection was performed using the Ion AmpliSeq Cancer Hotspot Panel v2, comprising 207 amplicons covering approximately 2,800 COSMIC mutations from 50 oncogenes and tumour suppressor genes. Data are analysed using Torrent Suite, Variant Caller and Ion Reporter. CHPv2 comprises hotspot regions within the following genes, using the reference transcripts as shown (where A of the ATG start site is designated 1). Exon numbering is transcript-specific; ABL1 (NM_005157.5,ex 4- 7); AKT1 (LRG_721t2, ex.3,5); ALK (LRG_488t1,ex 23,25); APC (LRG_130t1, ex 16); ATM (LRG_135t1,ex 8-9,12,17,26,34-36,39, 50,54-56,59,61,63); BRAF (LRG_299t1,ex 11,15); CDH1 (LRG_301t1,ex 3,8-9); CDKN2A (LRG_11t1, ex 2); CSF1R (NM_005211.3,ex 7,22); CTNNB1 (NM_001904.3,ex 3); EGFR (LRG_304t1,ex 3,7,15,18- 21); ERBB2 (LRG_724_t2, ex 19-21); ERBB4 (NM_5235.2,ex 3,4,6- 9,15,23); EZH2 (LRG_531t1,ex 16); FBXW7 (LRG_1141t1, 7,10-13); FGFR1 (LRG_993t1,ex 4,7); FGFR2 (LRG_994t1, ex 7,9,12); FGFR3 (LRG_1021t1, ex 7,9,14,16,18); FLT3 (LRG_457t1,ex 11,14,16,20); GNA11 (NM_002067.4,ex 5); GNAQ (NM_002072.4,ex 5); GNAS (NM_000516.5,ex 8-9); HNF1A (LRG_522t1,ex 3,4); HRAS (NM_001130442.2,ex 2-3); IDH1 (LRG_610t1, ex 4); IDH2 (LRG_611t2,ex 4); JAK2 (LRG_612t1,ex 14); JAK3 (LRG_77t1,ex 4,13,16); KDR (NM_002253.3,ex 6-7,11,19,21,26-27,30); KIT (LRG_307t1,ex 2,9-11,13-

15,17,18); KRAS (NM_004985.4,ex 2- 4); MET (LRG_622t1,ex 2,11,14,16,19); MLH1 (LRG_216t1,ex 12); MPL (LRG_510t1,ex 10); NOTCH1 (LRG_1122t1,ex 26,27,34); NPM1 (LRG_458t1,ex 11); NRAS (LRG_92t1,ex 2- 4); PDGFRA (LRG_309t1,ex 12,14,15,18); PIK3CA (LRG_310t1,ex 2,5,7,8,10,14,19,21); PTEN (LRG_311t1,ex 1,3,5- 8); PTPN11 (LRG_614t1,ex 3,13); RB1 (LRG_517t1,ex 4,6,10,11,14,17,18,20-22); RET (LRG_518t1,ex 10,11,13,15,16); SMAD4 (LRG_318t1,ex 3-6,8-12); SMARCB1 (LRG_520t1,ex 2,4,5,9); SMO (NM_005631.3,ex 3,5,6,9,11); SRC (LRG_1018t1, ex 14); STK11 (LRG_319t1, ex 1,4,6,8); TP53 (LRG_321t1, ex 2,4-8,10); VHL (LRG_322t1,ex 1-3). This assay has been validated with total genomic DNA of not less than 10ng and where there is at least 5% mutant DNA present. Below these cut-off levels, variants may not be consistently identified. This test does not detect large insertions, deletions, or duplications or genomic copy number variants. All variants of unknown significance have been excluded.

References

1. Ianevski, A., Giri, K. A., Aittokallio, T., 2020. SynergyFinder 2.0: visual analytics of multi-drug combination synergies. Nucleic Acids Research. gkaa216, https://doi.org/10.1093/nar/gkaa216

Supplementary Table 1. Details of CLL patients used in the BCR stimulation experiments. (Figure 3 and Figure 4).

CLL Research Consortium Patients			
**Patient ID**	**IgM +**	**Cytogenetics (FISH)**	**IgHV**
TJK0065	YES	13q-, +12	Unmutated
KRR0076	YES	Normal	Mutated
JGG0152	YES	Normal	Unmutated
TJK0875	YES	13q-	Mutated
KRR0667	YES	+12	Mutated
TJK0966	YES	13q-, +12	Mutated
KRR0112	YES	+12, 11q-	Unmutated
JGG0303	YES	Normal	Unmutated
JGG0183	YES	Unknown	Unmutated
JGG0281	YES	Normal	Unmutated
MJK1009	YES	13q-, +12, 17p-	Unmutated
TJK0270	YES	+12	Mutated
MJK0844	YES	13q-, 17p-	Mutated
JCB0755	YES	Normal	Mutated
TJK0834	YES	13q-	Unmutated
JCB0252	YES	13q-, 17p-	Unmutated
TJK0895	YES	Normal	Mutated
JRB0395	YES	13q-	Mutated
WGW0167	YES	13q-, 11q-	Unmutated
MJK1290	YES	13q-	Unmutated
TJK0705	YES	13q-	Unmutated
JRB0122	YES	13q-, 11q-, 17p-	Unmutated
TJK0917	YES	13q-	Unmutated
KRR0681	YES	13q-	Mutated
WGW0010	YES	13q-	Unmutated
TJK1047	YES	13q-, 11q-	Unmutated
KRR1088	YES	13q-	Mutated
KRR1154	YES	13q-, 11q-	Mutated
JCB0952	YES	13q-	Mutated
JRB0532	YES	13q-	Mutated
TJK1580	YES	17p-, 13q-, tet11q	Unknown

Barts Cancer Institute Patients			
**Patient ID**	**IgM +**	**Cytogenetics (FISH)**	**Indolent/progressive* (Binet stage)**
5959	YES	13q-	Indolent (A)
6966	YES	Unknown	Indolent (A)
8002	YES	+12	Indolent (A)
6799	YES	13q-	Indolent (A)
9352	YES	Normal	Indolent (A)
9528	YES	13q-	Indolent (A)

8183	YES	Unknown	Indolent (A)
5930	YES	13q	Indolent (A)
7581	YES	13q-	Indolent (A)
7655	YES	13q-	Indolent (A)
T0602	YES	13q-	Indolent (A)
9746	YES	13q-	Progressive: LNs, Spln (B)
8549	YES	13q-, 11q-	Progressive: Spln, BMF, LDT (C)
6083	YES	+12; 14q rearr.	Progressive: LNs, LDT (B)
10550	YES	11q-	Progressive: LNs, LDT (B)
9024	YES	17p-, +12,	Progressive: LNs, Spln, BMF (C)
8368	YES	+12; 13q-	Progressive: LNs, Spln, BMF (C)
8598	YES	13q-	Progressive: LNs (B)
9491	YES	17p-	Progressive: Spln, BMF, LDT (C)
8921	YES	17p-	Progressive: LNs, Spln, LDT (B)
8138	YES	13q-	Progressive: LNs, BMF, LDT (C)
9854	YES	+12	Progressive: LNs, Spln, LDT (B)
11270	YES	13q	Progressive: LNs, BMF (C)
11018	YES	+12	Progressive: LNs, BMF, LDT (C)
T2596	YES	13q-	Progressive (B)
R9975	YES	17p-; +12	Progressive: LDT, LNs, Spln (B)
T7980	YES	13q-	Progressive: BMF, LNs, Spln (C)
T1799	YES	17p-; 11q-	Progressive: LNs, LDT; BMF (C)
T4440	YES	17p-	Progressive (B)
T3831	YES	17p-; 13q-	Progressive: LNs, WBC, BMF (C)
R8854	YES	17p-	Progressive: Spln (B)
T6536	YES	17p-	Progressive: LNs, WBC, Spln (B)
T6767	YES	17p-	Progressive: Spln; BMF, WBC (C)
*LNs = lymphadenopathy; Spln = splenomegaly; BMF = bone marrow failure; LDT = rapid lymphocyte doubling time			

Supplementary Table 2. Details of CLL patients used in the experiments with no BCR stimulation (Figure 2 and Figure 5).

CLL Research Consortium Patients		Barts Cancer Institute Patients	
**Patient ID**	**17p- status**	**Patient ID**	**17p- status**
JGG0033	17p- (95.5%)	T6767	17p- (92%)
JRB0761	17p- (99%), 13q-	T3831	17p- (86%), 13q-
IWF0001	17p- (92%), 13q-	T6536	17p- (72%)
JRB0697	17p- (82.5%), tri 12	R4578	tri 12, 14q rearr.
JRB0633	17p- (94%), tri 12	T2596	13q-
JRB0317	17p- (79%), 13q-	R5989	17p- , tri 12
JRB0318	17p- (88%), 13q-	T1799	17p- (75%), 11q
MJK0617	17p- (93.5%), 13q-	T0605	17p- (86%)
JCB0252	17p- (90%), 13q-	T1615	17p-, 13q-
JRB0475	17p- (88.5%), 13q-	T4140	17p- (41%)
JRB0769	17p- (99%)	R6274	17p-, tri 12
TJK1200	13q-	R9793	17p- (15%), 13q-, 11q-
MJK1654	13q-	R9975	17p- (60%), tri 12
JRB0649	17p- (84.5%)	R8854	17p-
JRB0693	17p- (91.5%)	R6695	13q-
JRB0717	17p- (83.4%), 13q-, 11q-	T8957	normal
JRB1028	17p- (94.5%), tri 12	R9559	13q-
TJK1580	17p- (76%), 13q-, tet 11q, t(11;14) tet	T0068	tri 12
KRR1158	13q-	T8959	13q-
JRB0528	13q-	R9078	13q-
KRR1112	11q-, 13q-	T0602	13q-
JRB1165	17p- (85%), 13q-, tri 12	T4440	17p- (47%)
TJK1254	11q-	T5110	17p- (47%), tri 12
JCB1318	17p- (89%), tri 12	T7940	17p-
JRB0690	17p- (95.5%)	T9749	17p-, 13q-
JRB0103	17p- (99%), 13q-, 11q-		
TJK1212	13q-		
TJK0761	13q-		
MJK1009	17p- (85%), 13q-, t12		
JRB0362	17p- (97.5%), 13q-		
TJK0553	17p- (93%)		
JRB0122	17p- (82%), 13q-, 11q-		
KRR1104	13q-		
WGW0122	13q-		
JRB0470	17p- (84%)		
JRB0172	17p-		

Supplementary Table 3 (next page). Ion AmpliSeq Cancer Hotspot Panel v2 sequencing results of 17p-del CLL samples shown in bold letters (first 26 in red and orange) and control non-17p-del CLL samples (last 8 shown in green). 'Good GLC consumers' are underlined and shown in red at the top of the table. TP53 mutations known to alter metabolism are highlighted in yellow.

Sample ID	Cytogenetics (% 17p-)	TP53 mutation	VAF (*TP53* mut)	Type of *TP53* mutation	Other mutation	VAF
**JCB0252**	17p-(90%), 13q-	c.581T>G p.(Leu194Arg)	100%	missense	nd	-
**JRB0633**	17p-(94%), Tris12	c.310dup p.(Gln104Profs*45)	100%	frameshift	BRAF c.1786G>C p.(Gly596Arg)	4%
**JRB0318**	17p-(88%), 13q-	c.614A>T p.(Tyr205Phe)<br>c.524G>A p.(Arg175His)	91%<br>4%	missense<br>missense	nvd	-
**T6536**	17p- (72%)	c.643A>C p.(Ser215Arg)	18%	missense	nvd	-
**T6767**	17p- (92%)	nvd	-	-	APC c.3949G>C p.(Glu1317Gln)	52%
**T9975**	17p- (60%); Tri 12	c.818G>A p.(Arg273His)	99%	missense	nvd	-
**JRB0717**	17p- (83.4%), 13q-, 11q-	c.794T>C p.(Leu265Pro)	90%	missense	nvd	-
**TJK1580**	17p- (76%), 13q-, tet 11q, t(11;14) tet	nvd	-	-	nvd	-
**JCB1318**	17p- (89%), tri 12	c.838A>G p.(Arg280Gly)	74%	missense	nvd	-
**MJK1009**	17p- (85%), 13q-, t12	c.659A>G p.(Tyr220Cys)	99%	missense	nvd	-
**JRB0122**	17p- (82%), 13q-, 11q-	c.451C>T p.(Pro151Ser)	98%	missense	nvd	-
**T4140**	17p- (41%)	c.827C>A p.(Ala276Asp)	91%	missense	nvd	-
**T3831**	17p- (86%), 13q- (76%)	c.1010G>C p.(Arg337Pro)	100%	missense	nvd	-
**T4440**	17p- (47%)	c.380C>T p.(Ser127Phe)	7%	missense	nvd	-
**IWF0001**	17p- (92%), 13q-	c.733G>A p.(Gly245Ser)	94%	missense	nvd	-
**JRB0697**	17p- (82.5%), tri 12	c.637C>T p.(Arg213*)	72%	nonsense	nvd	-
**JGG0033**	17p- (95.5%)	c.376-3C>G p.	100%	splice site (VUS)	nvd	-
**JRB0317**	17p- (79%), 13q-	c.535C>T p.(His179Tyr)	100%	missense	nvd	-
**JRB0649**	17p- (84.5%)	c.736A>C p.(Met246Leu)<br>c.701A>G p.(Tyr234Ser)<br>c.1039G>A p.(Ala347Thr)<br>c.395A>G p.(Lys132Arg)	37%<br>29%<br>12%<br>12%	missense<br>missense<br>missense<br>missense	nvd	-
**JRB0690**	17p- (95.5%)	nvd	-	-	PTEN c.987_990del p.(Asn329Lysfs*14)	43%
**JRB0761**	17p- (99%), 13q-	c.393_395del p.(Asn131del)	100%	deletion	nvd	-
**JRB0769**	17p- (99%)	c.524G>A p.(Arg175His)	98%	missense	nvd	-
**MJK0617**	17p- (93.5%), 13q-	c.731G>A p.(Gly244Asp)	100%	missense	nvd	-
**R8854**	17p- (ND)	c.844C>G p.(Arg282Gly)	99%	missense	nvd	-
**R9749**	17p- (ND), 13q-	nvd	-	-	nvd	-
**T7940**	17p- (ND)	c.469G>T p.(Val157Phe)	97%	missense	nvd	-
TJK1254	11q-	nvd	-	-	nvd	-
R2596	13q-	c.743G>A p.(Arg248Gln)	3%	missense	nvd	-
R4578	t12; 14q rearr.	nvd	-	-	BRAF c.1803A>T p.(Lys601Asn)	19%
T8957	Normal	nvd	-	-	nvd	-
R9559	13q-	nvd	-	-	nvd	-
T0068	Tri 12	nvd	-	-	BRAF c.1397G>T p.(Gly466Val)	49%
TJK1200	13q-	nvd	-	-	nvd	-
JRB0528	13q-	nvd	-	-	nvd	-

nvd = no variant detected; VUS = variant of unknown significance; ND = not determined

Supplementary Figure 1

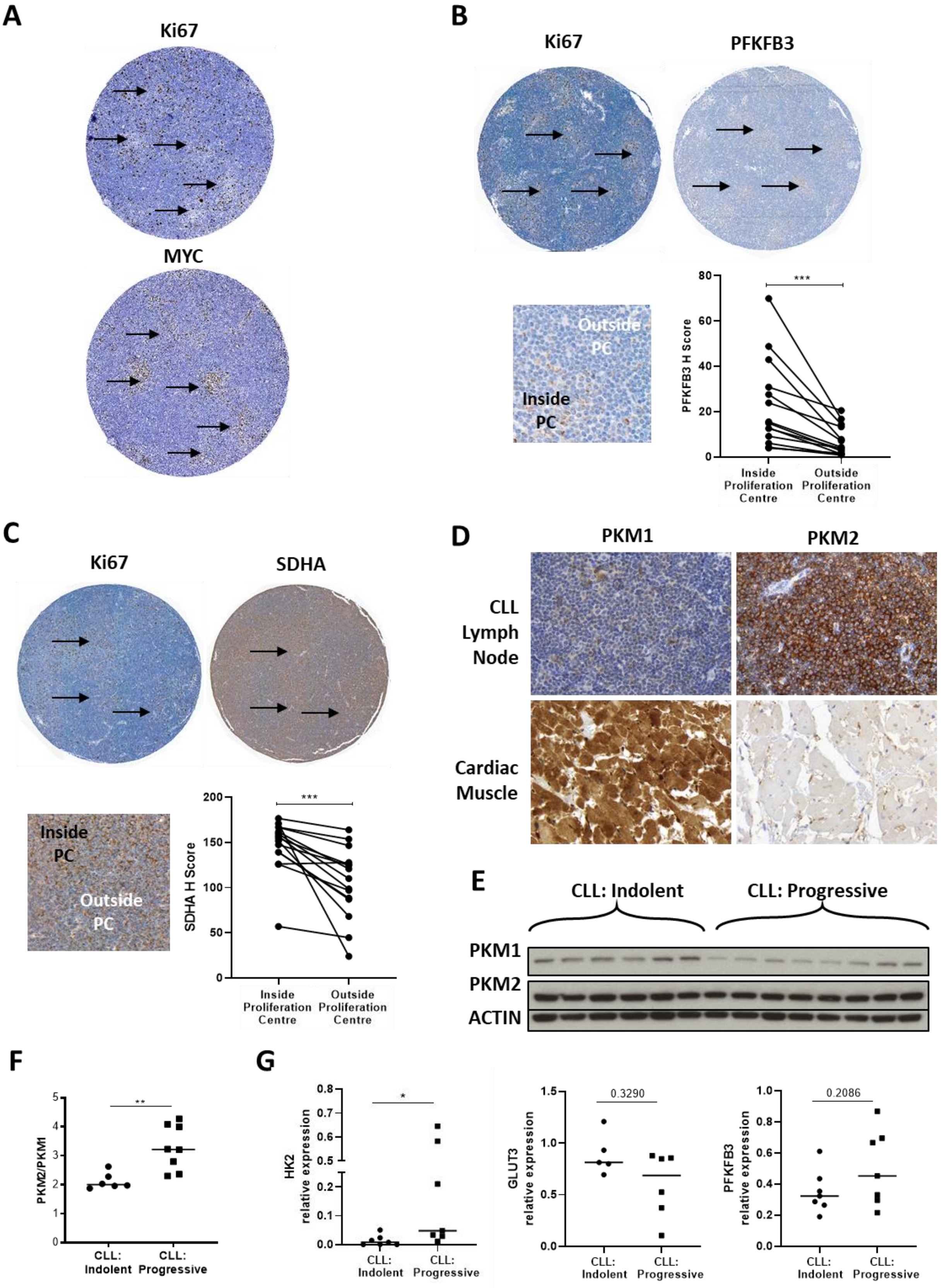

Supplementary Figure 1. Increased expression of MYC and metabolic markers within proliferation centers in CLL lymph nodes. (A) Representative images of Ki67 and MYC showing increased expression of MYC within proliferation centers (paler areas with an increased proportion of larger $Ki67^{+}$ cells; arrowed). (B, C) The expression of PFKFB3 (B) and SDHA (C) was assessed in lymphoid tissue CLL cells by immunohistochemistry. Representative images of whole cores are shown. Detailed images show higher expression of Ki67 (B, C), PFKFB3 (B) and SDHA (C) in CLL cells within proliferation centers (arrowed) (n = 14 for PFKFB3 and n=15 for SDHA). (D) Representative images showing very high expression of PKM2 but low expression of PKM1 in CLL lymph nodes. In contrast, the reverse pattern was seen with cardiac muscle (stained on the same tumor microarray under identical staining conditions). (E, F) Immunoblot comparing the expression of PKM1 and PKM2 in circulating CLL cells in patients with indolent (Rai stage 0/I; Binet stage A) or progressive (Rai stage II-IV; Binet stage B/C) disease). The fall in PKM1 with disease progression was reflected in an increase in the PKM2:PKM1 ratio (n=6 for indolent disease and n=8 for progressive disease). (G) Increased expression of HK2 in patients with progressive versus indolent disease (described as above) with no change in GLUT3 and PFKFB3 (n=7 for both indolent and progressive CLL for HK2 and PFKFB3; n=5 for indolent CLL and n=6 for progressive CLL for GLUT3).

Supplementary Figure 2

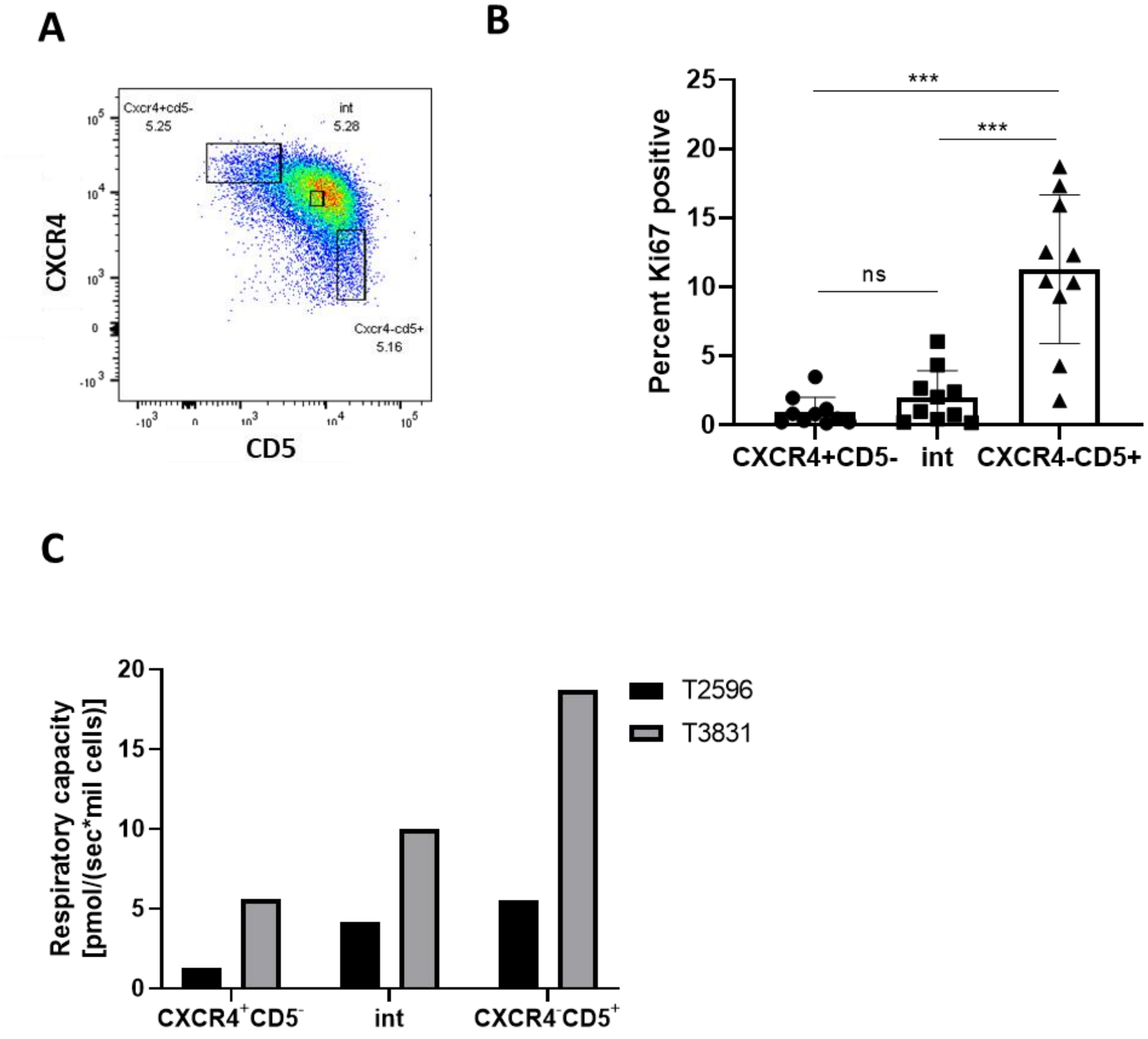

Supplementary Figure 2. Circulating CXCR4$^-$CD5$^+$ CLL cells exhibit increased proliferation. (A) Representative gating of primary CLL cells stained with CXCR4 and CD5 antibodies. CXCR4$^+$CD5$^-$, intermediate (int) and CXCR4$^-$CD5$^+$ fractions were gated as ≈ 5 % of the whole population. (B) Primary CLL cells were labelled with CXCR4 and CD5 antibodies as in (A), fixed and further stained with Ki67 to determine the percentage of proliferating cells (Ki67 positive) in the fractions gated as shown in (A) (n=10). (C) Oxygen consumption was assessed in two primary CLL samples sorted into fractions as shown in (A).

Supplementary Figure 3

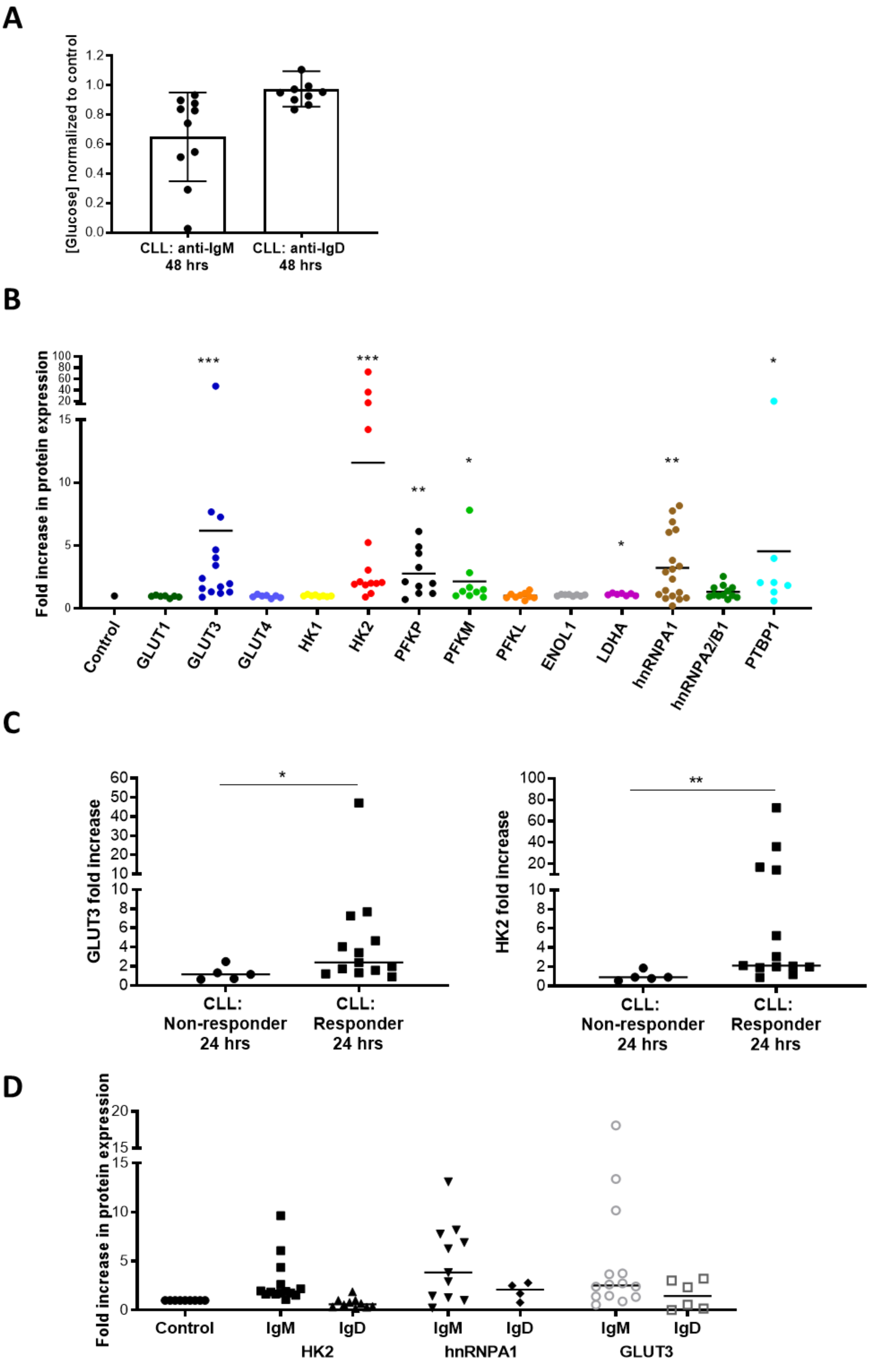

Supplementary Figure 3. BCR-stimulation of CLL cells induces the expression of glycolytic enzymes and transporters. (A) Anti-IgD stimulation (n = 10) had a negligible effect on glucose uptake with the residual concentration of glucose comparable to media only control. (B) CLL cells were stimulated by anti-IgM or isotype control for 24 hours and the expression of a panel of glycolytic enzymes was assessed by immunoblot and densitometry. GLUT3 (n = 14), HK2 (n = 14), PFKP (n = 10), PFKM (n = 9), hnRNPA1 (n = 18) and PTBP1 (n = 7) all had low basal expression that was induced by anti-IgM stimulation. ENOL-1 (n = 8) and LDHA (n = 7) were constitutively expressed with anti- IgM stimulation having no/a minimal effect. Anti-IgM stimulation had no effect on GLUT1, GLUT4, HK1, PFKL, and hnRNPA2/B1. (C) Samples that exhibited robust calcium flux after anti-IgM stimulation (n = 13) had a significantly higher fold change increase in the expression of GLUT3 and HK2 compared to non-responders as assessed by western blot and densitometry (n = 5). (D) The relative effects of anti-IgM and anti-IgD stimulation on the expression of HK2, hnRNPA1 and GLUT3 were assessed. Anti-IgD stimulation had no effect on HK2 and had a weaker effect on the induction of hnRNPA1 and GLUT3.

Supplementary Figure 4

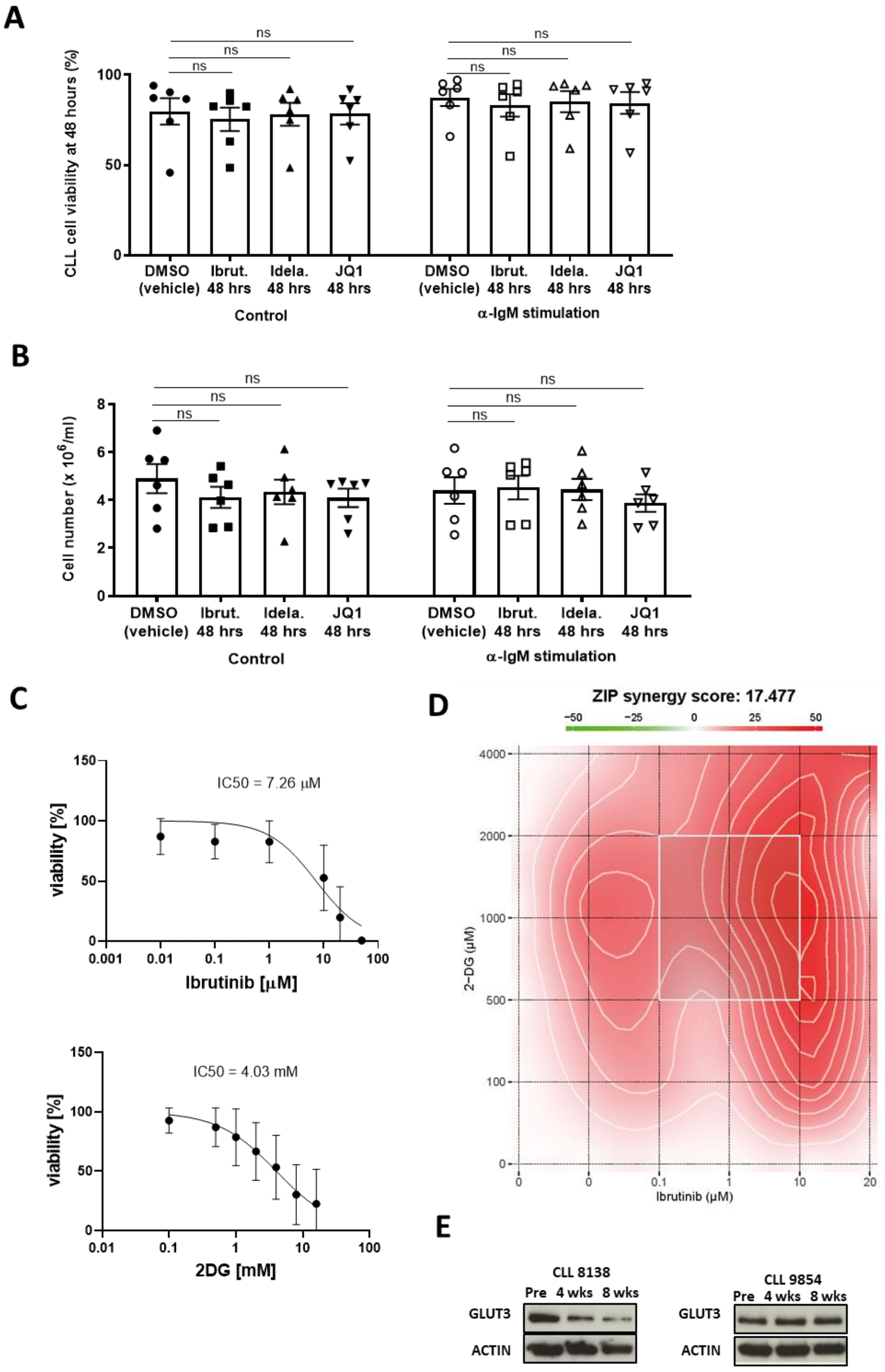

Supplementary Figure 4. Inhibition of MYC and BCR-signaling in anti-IgM stimulated CLL cells. (A, B) The impact of ibrutinib, idelalisib and JQ1 on CLL cell viability and number after 48 hours culture was assessed by Trypan Blue exclusion with either anti-IgM stimulation or control (n=6). (A) Inhibition of MYC and BCR-signaling had no significant impact on cell viability after 48 hours culture either with anti-IgM stimulation or control. (B) Similarly, inhibition of MYC and BCR-signaling had no significant impact on cell number after 48 hours culture either with anti-IgM stimulation or control. (C) Primary CLL cells were treated with increasing concentrations of Ibrutinib or 2-DG for 48 hours and their IC50 was calculated. Ibrutinib; IC50=7.26µM (n=14) and 2-DG; IC50=4.03mM (n=13) (D) Primary CLL cells (n=11) were treated with increasing concentrations of Ibrutinib and 2-DG individually and in combination according to 6x6 matrix as shown in the synergy map generated using SynergyFinder. The synergy map highlights synergistic areas in red, with the most synergistic concentrations determined for 10 µM Ibrutinib and 1 mM 2-DG. (E) Representative blots of a patient (CLL 8138) who had significant reduction in GLUT3 with ibrutinib treatment and another (CLL 9854) where there was no change.

SupplementaryFigure 5

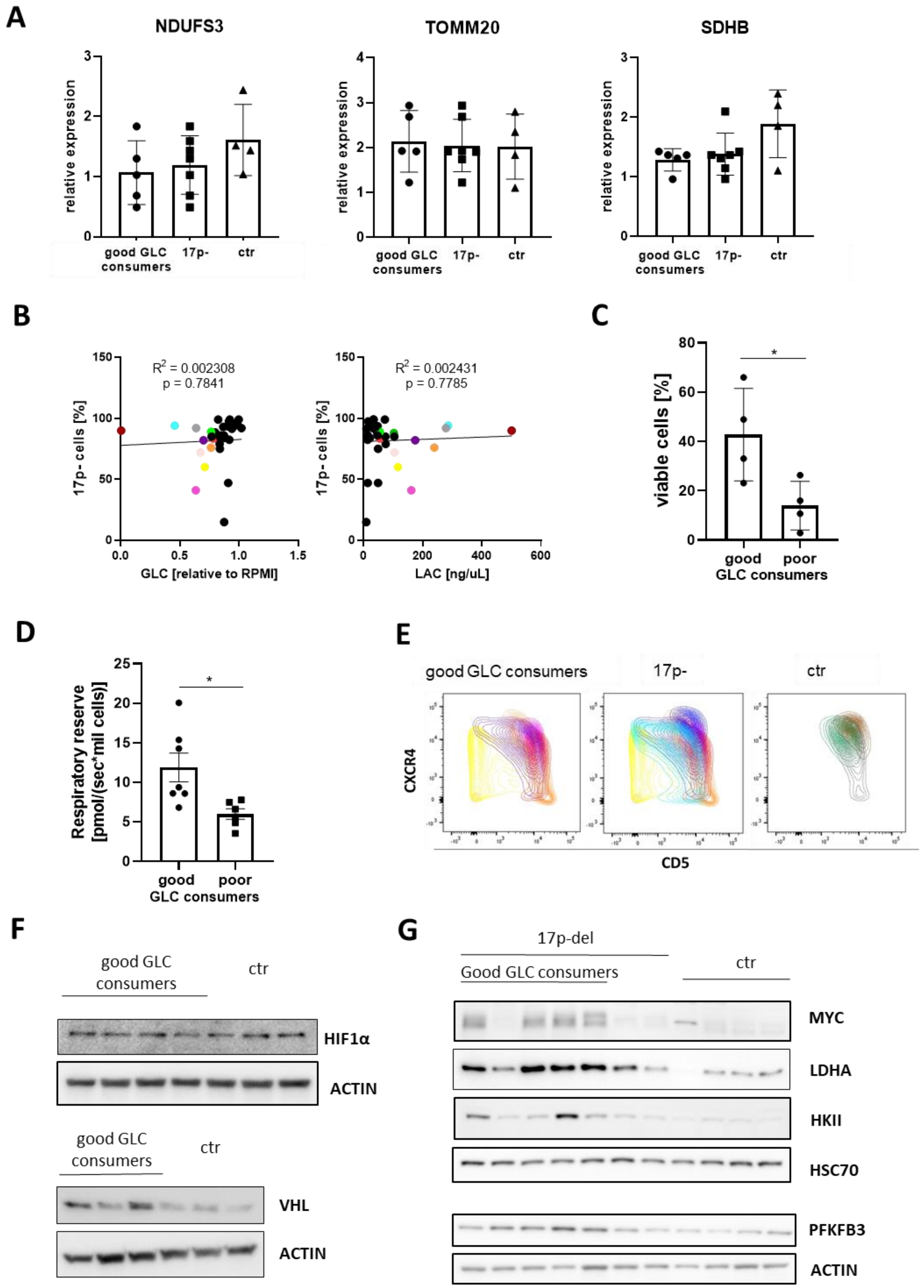

Supplementary Figure 5. 17p- ‘Good GLC consumers’ characterization vs non-17p- control CLL. (A) No significant change in the expression of NDUFS3, TOMM20 or SDHB was observed between 17p- (n=7) and control non-17p- (n=4) CLL samples as assessed by western blot and densitometry. ‘Good GLC consumers’ (n=5) shown are extracted from the 17p- samples assessed. (B) No correlation was observed between the percentage of 17p- cells and spontaneous glucose consumption or lactate production (data extracted from those shown in Figure 5A,B). Good GLC consumers are highlighted in colors. (C) 17p- primary CLL cells were treated with combination of 10 µM ibrutinib and 1 mM 2-DG for 48 hours and the level of apoptosis determined by Annexin V staining. Good glucose consumers (n=4) were more resistant than poor glucose consumers (n=4). (D) Good glucose consumers (n=7) have higher respiratory capacity than poor glucose consumers (n=6) (data extracted from those shown in Figure 5F). (E) 17p- (n=7) and control non-17p- (n=3) CLL cells were labelled with CXCR4 and CD5 antibodies and assessed on FACS for their CXCR4/CD5 profile. Good GLC consumers (n=5) shown are extracted from the 17p- samples group. (F) Constitutive expression of HIF-1α and VHL in 17p- good GLC consumers (n=4 for HIF-1α; n=3 for VHL) compared to non-17p- controls (n=3). (G) Representative immunoblot of 17p- (n=12) and non-17p- controls (n=8) showing increased expression of MYC, LDHA and HKII in 17p-del CLL samples.
